# Prevalence and associated factors of maternal birth trauma following vaginal delivery at University of Gondar Comprehensive Specialized Hospital, North-West Ethiopia, 2022

**DOI:** 10.1186/s12884-024-06635-4

**Published:** 2024-06-27

**Authors:** Nigat Amsalu Addis, Demelash Abraham, Mihret Getnet, Alehegn Bishaw, Zelalem Mengistu

**Affiliations:** 1 Department of Gynecology and Obstetrics, School of Medicine, College of Medicine and Health Sciences, University of Gondar, Gondar, Ethiopia; 2 Department of Human Physiology, School of Medicine, College of Medicine and Health Sciences, University of Gondar, Gondar, Ethiopia; 3 Department of Epidemiology and biostatistics, Institute of public Health, College of Medicine and Health Sciences, University of Gondar, Gondar, Ethiopia; 4 Department of Reproductive and Child Health, Institute of public Health, College of Medicine and Health Sciences, University of Gondar, Gondar, Ethiopia; 5 Department of Obstetrics and Gynecology, Division for Clinical Medicine, University of Global Health Equity, Kigali, Rwanda

**Keywords:** Cervical laceration, OASIs, Perineal trauma, Vaginal delivery, Ethiopia

## Abstract

**Background:**

Maternal injury with any form of perineal trauma following vaginal delivery is very common which ranges globally from 16.2 to 90.4%. The frequency of Obstetric anal sphincter Injuries and the incidence of cervical laceration increases rapidly. However, in Ethiopia, there is limited evidence on the prevalence of maternal birth trauma and its determinant factors after vaginal delivery.

**Objective:**

To assess the magnitude and associated factors of Maternal Birth Trauma after vaginal delivery at University of Gondar Comprehensive Specialized Hospital, Gondar, North-West Ethiopia, 2022.

**Methods:**

An Institution based cross-sectional study was conducted among mothers with singleton vaginal delivery at University of Gondar Comprehensive Specialized Hospital from 9th May to 9th August 2022 among 424 study participants. Pre-tested semi-structured questioner was utilized. Epi-Data version 4.6 was used for data entry and exported to SPSS version 25 for data management and analysis. To identify the determinant factors, binary logistic regression model was fitted and variables with p-value < 0.2 were considered for the multivariable binary logistic regression analysis. In the multivariable binary logistic regression analysis, Variables with P-value < 0.05 were considered to have statistical significant association with the outcome variable. The Adjusted Odds Ratio (AOR) with 95% CI was reported to declare the statistical significance and strength of association between Maternal Birth Trauma and independent variables.

**Results:**

A total of 424 mothers who delivered vaginally were included. The mean age of participants was 26.83 years (± 5.220 years). The proportion of birth trauma among mothers after vaginal delivery was47.4% (95%CI: 43.1, 51.7). Of different forms of perineal trauma, First degree tear in 42.8%, OASIs in 1.5% and Cervical laceration in 2.5% study participants. In the multivariable binary logistic regression analysis being primiparous (AOR = 3.00; 95%CI: 1.68, 5.38), Gestational age ≥ 39 weeks at delivery (AOR = 2.96; 95%CI: 1.57, 5.57), heavier birth weight (AOR = 12.3; 95%CI: 7.21, 40.1), bigger head circumference (AOR = 5.45; 95%CI: 2.62, 11.31), operative vaginal delivery (AOR = 6.59; 95%CI: 1.44, 30.03) and delivery without perineum and/or fetal head support (AOR = 6.30; 95%CI: 2.21, 17.94) were significantly associated with the presence of maternal birth trauma.

**Conclusion and recommendation:**

Maternal birth trauma following vaginal delivery was relatively high in this study. Prim parity, gestational age beyond 39 weeks at delivery, heavier birth weight, bigger head circumference, operative vaginal delivery and delivery without perineum and/or fetal head supported were factors affecting perineal outcome. The Ministry of Health of Ethiopia should provide regular interventional training as to reduce maternal birth trauma.

## Introduction

### Statement of the problem

Maternal birth traumas following vaginal delivery are very common which contribute to significant maternal morbidity and even to death [1]. Prevention, early detection with prompt and effective management minimizes maternal morbidity and prevents many gynecological problems from developing later in life [2–4]. The perineum and vagina should be thoroughly examined for evidence of injury following delivery of the placenta. The cervix should be examined if there is significant bleeding or following operative vaginal delivery [5].

The international Consultation on Incontinence (ICI) and Royal College of Obstetricians and Gynecologists (RCOG) adopts classifications of Obstetric Anal Sphincter injuries (OASIs) in to Four common lower genital tract injuries are perineal, vulvar, vaginal, and cervical [6].

Maternal injury with any form of perineal trauma following vaginal delivery is very common [7–10]. Obstetric anal sphincter Injury is a serious complication of labor and delivery and the percentage of OASI worldwide range from 0.1 to 25% [11–16]. This significant variation is attributed to the different obstetric care centers, the time when clinical investigation was done, parity, ethnicity, use of instrumental delivery, episiotomy use, maternal position during active pushing, duration of second stage of labor and other obstetric factors. Women with previous anal sphincter lacerations are 3 to 5 fold increased risk for subsequent sphincter laceration compared with women with prior vaginal delivery without sphincter laceration, but recurrence is not predictable using pre-delivery anal physiology testing [17].

Cervical laceration is known cause of postpartum hemorrhage (PPH) [18]. The prevalence of Cervical Laceration ranges from 0.2 to 1.1% [19–22]. Cerclage placement is significantly associated with an increased risk of cervical laceration with a 3.7 fold in nulliparous women and 12.7 fold increased risk in multiparous women [22]. Induction of labor contributes about a 3.1 fold increase in the rate of cervical lacerations [21]. Recurrent laceration is 4.9% in those having history of cervical lacerations in the previous pregnancy. History of intrapartum cervical laceration is an independent risk factor for recurrent cervical lacerations, Cesarean Delivery (CD), preterm delivery, and severe perineal lacerations in the subsequent pregnancy [20].

Some of the known factors associated with maternal birth trauma following vaginal delivery incudes parity, gestational age, fetal birth weight, vaginal breech delivery, Vaginal birth after Cesarean, operative vaginal delivery, maternal position during active pushing, duration of second stage of labor, previous anal sphincter lacerations, cervical Cerclage, Induction and Augmentation of labor [12, 14, 23].

Antepartum perineal massage (APM), Mediolateral episiotomy, Epidural analgesia use during instrumental delivery, lateral position during active pushing and delivery, slowing the delivery of the infant’s head and instructing the mother not to push while the head is being delivered are associated with reduced risk of maternal birth trauma [23–27].

Complications following maternal birth injuries include recurrent anal sphincter injury [17], postpartum perineal pain [28], postpartum chronic pain [4], postpartum hemorrhage (PPH) [18], dyspareunia and sexual dysfunction [29, 30], anorectal complaints and urinary incontinence which affect overall quality of women’s life [30–32].

A study from United Kingdom shows perineal pain affects 92% of mothers and episiotomy causes more perineal pain than spontaneous second degree tears [28]. Another study from United States shows women with intact perineum after vaginal delivery reported the best overall sexual function than those with perineal trauma [29].

A study from Netherlands shows that pain during or after intercourse was high in women with history OASIs of more than two decades than women without OASIs (29% of cases versus 13% of controls). Fecal incontinence during intercourse was increased by thirteen fold of cases than controls [30].

More than one half of the women had new onset of urinary incontinence (UI) after delivery and reported several lifestyle modifications to prevent leakage in United States (34). From one systematic review and meta-analysis from Netherlands, the prevalence of UI is nearly the same level as in the third trimester of pregnancy at 1 year post-partum (32%). Stress UI is the most prevalent type (54%) [32].

There is only one research done in sub-Saharan African countries showing the magnitude of perineal trauma and associated factors despite having the higher burden of maternal birth injury following vaginal delivery. There is no article published in Ethiopia, where there is long lived trend of delivery in Dorsolithotomy position and higher magnitude perineal trauma from day to day clinical observations. Hence, this study will flourish the ground in this nation to determine the magnitude of maternal birth canal injuries following vaginal delivery and factors attributed to it.

## Methods and materials

### Study period and setting

The study was conducted in UoGCSH, found in Gondar town, which is the capital town of the Central Gondar administrative zone, located 741kms northwest of Addis Ababa. UoGCSH is one of the biggest tertiary level referral and teaching hospitals in the Amhara Regional State.

According to records from the hospital’s information center, every year more than 200,000 people visit the hospital which serves as referral hospital for more than 7 million people in the surrounding catchment area with varying climatic and geographical distributions. The hospital has more than 1000 beds of which nearly 200 beds are allocated to Obstetrics and Gynecology services. UoGCSH department of Obstetrics and Gynecology had one labor and delivery ward with nine beds in the first stage room, six delivery couches in the second stage room along with two emergency operating rooms, three postpartum maternity wards, one high risk ward, one gynecology ward, one Fistula center, Michu clinic, four Gynecologic OPD, and four Antenatal care (ANC) clinics. It has also 3 outreach centers destined for Comprehensive Emergency Obstetric Care (CEmOC). It has 24 Obstetricians and Gynecologists and 73 residents from first to fourth year of residency, 80 medical intern doctors, and 165 professional midwives. There were 3267 singleton, 63 twin and 1 triplet deliveries, a total of 3331 deliveries, recorded over three months (93 days) from 9th May to 9th August, 2022. Of all 2354 were spontaneous vaginal delivery, 832 by cesarean section, 50 were by operative vaginal delivery, 15 cesarean hysterectomy, 13 assisted breech delivery and 3 destructive delivery. Of all 2407 vaginal deliveries with cephalic presentation (Spontaneous vaginal deliveries + OVD + Destructive Deliveries), 1272 (≈ 53%) had reliable date or early milestone from which it is calculated to be ≥ 37W0D.

### Study design

Institution based cross-sectional study was conducted to determine the magnitude of maternal birth trauma and determinant factors after vaginal delivery at University of Gondar Comprehensive Specialized Hospital (UoGCSH).

### Source population

All mothers who gave birth vaginally at UoGCSH.

### Study population

All mothers who delivered vaginally at UoGCSH meeting the eligibility criteria during data collection time from 9th May to 9th August 2022.

### Eligibility criteria

All mothers who had singleton vaginal delivery at UoGCSH from during data collection were considered; whereas women with preterm delivery and malpresentation were excluded.

### Sample size determination

The sample size was estimated using single population proportion formula, n = (Zα/2)^2^ P (1-P)/δ2. By considering 95% level of confidence, 5% margin of error with assumption of 50% as the population proportion of women with birth trauma and 10% added for non-response rate.

Where;

n = Sample size.

Z = the standard normal deviation at 95% confidence interval; =1.96.

P = expected proportion of women with birth trauma, Hence, there is no reasonable estimate, and then 50% (0.5) will be used.

d = margin of error that can be tolerated, 5% (0.05).

$$n = {\left( {Z\alpha /2} \right)^2}P{\text{ }}\left( {1 - P} \right)/{\text{ }}{\delta ^2}$$

$$= {\left( {1.96} \right)^2}X{\text{ }}0.5\left( {1 - 0.5} \right)/{\left( {0.05} \right)^2}$$

*n* = 384.16 ≈ 385 and 10% non-response rate = 38.5 ≈ 39.

The final sample size was 424.

### Sampling technique

**Fig. 1:**
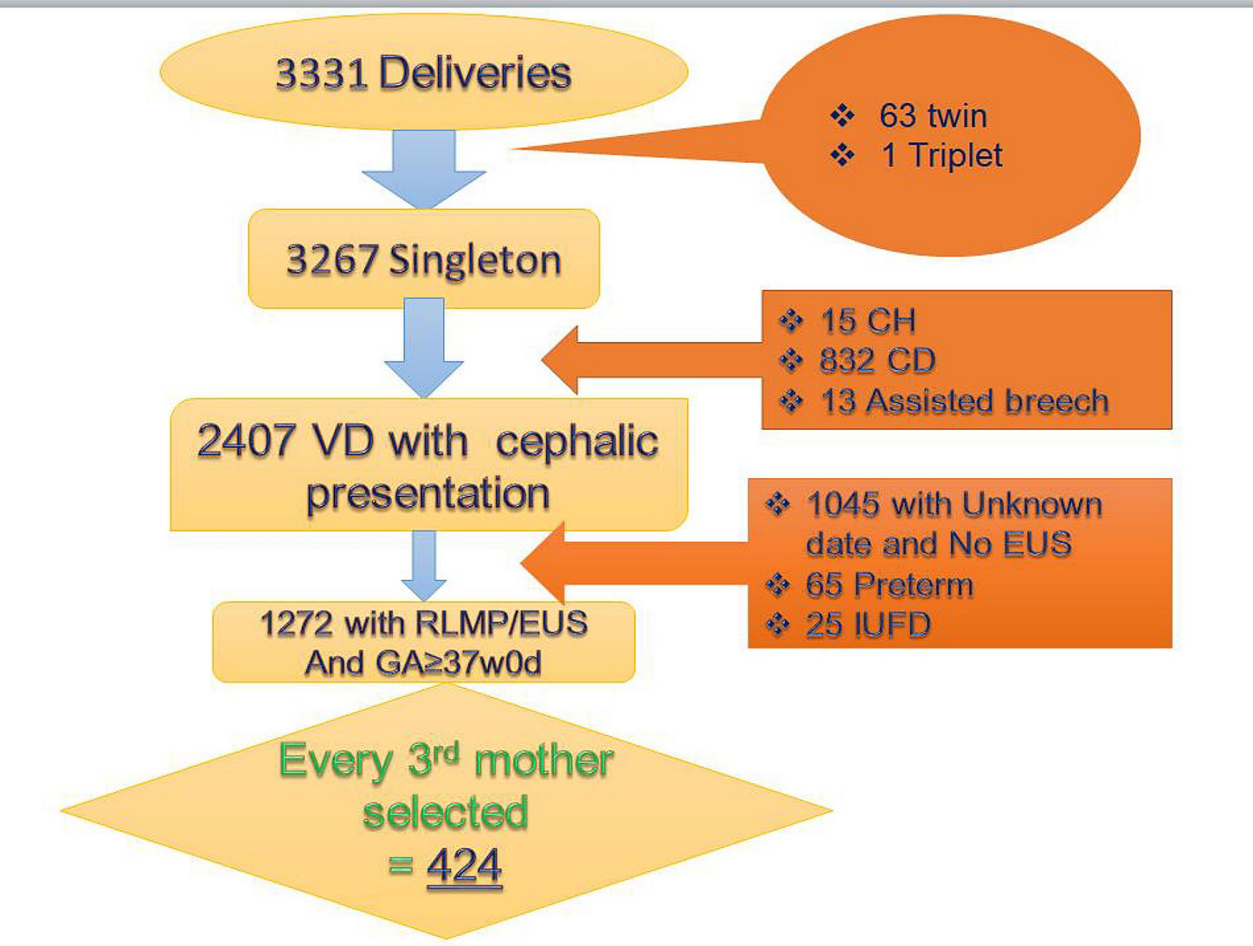
Sampling procedure used to select study participants on prevalence and associated factors of Maternal Birth traumas after vaginal delivery from 9th May to 9th August, at UoGCSH, 2022

A total of 2407Mothers with vaginal (Spontaneous, OVD and destructive) delivery of all 1272 were term pregnancy from reliable date or early ultrasound identified in the study period and 424 participants were selected using simple random sampling technique (Fig. 1).

### Data collection procedures

Data collection tools were pre-tested semi-structured questioner, data extraction checklist and physical examination. It was prepared in English and translated to Amharic Version for variables which need patient interview. The data collectors were two professional midwifes and five OBGYN residents. Onsite training was given for data collectors on the methods of collecting data through interview, data extraction from delivery summery sheet and medical records and physical examination. Neonatal head circumference was measured within 24 h of delivery. The ethical aspect in keeping the confidentiality of their information was another focus of the training.

### Study variables

#### Dependent variables

It was coded as 1 “Yes”; if there was Maternal Birth Trauma and 0 “No” for the absence of birth trauma.

#### Independent variables

Sociodemographic factors (age, marital status, residence, level of education, occupation, predominant work position and average monthly income), obstetric history (parity, pregnancy status, gestational age and number of ANC contacts), pushing in the 2nd Stage, maternal position during pushing, digital stretching before crowning, maternal position during delivery, type of delivery, delivery technique, indicated episiotomy done, fetal head position, total duration of labor, duration of second stage, induction/augmentation of labor, birth attendant, birth weight, neonatal sex and neonatal head circumference.

### Operational definition

#### Maternal birth trauma

is injury to the birth canal and/or perineum during vaginal delivery attributed to the labor and delivery process and needs repair due to large tissue gap or bleeding which includes at least one of the following; cervical lacerations, vaginal wall laceration, episiotomy extension and perineal tears [33].

#### Birth weight

is the first weight of the baby, taken just after being born rounded to the nearest grams.

#### Head circumference

Head circumference is a measurement of a child’s head around its largest area. It measures the distance from above the eyebrows and ears and around the back of the head.

#### Duration of labor

the total duration in hours from onset of true labor to delivery of the baby.

#### Duration of second stage of labor

the total time (in minutes) elapsed from full cervical dilation to delivery of the baby.

#### Severe perineal trauma

Involves third and/or fourth degree obstetrics perineal tears.

#### Obstetrics perineal tears

can be classified using the latest version adopted by RCOG and ICI.

#### First-degree

Injury to the perineal skin.

#### Second-degree

Injury to the perineum involving the perineal muscles, but not involving the anal sphincter.

**Third-degree**: Injury to the perineum involving the anal sphincter complex: 3 A: <50% of the EAS thickness torn, 3B: >50% of the EAS thickness torn and 3 C: both the EAS and the IAS torn.

#### Fourth degree

injury to the perineum, anal sphincter complex and rectal mucosa.

### Data quality control

Training was given to the data collectors and supervisors and pretest done on 5% [17] of study participants and corrected. Supervision was done for the data collectors by the supervisors and principal investigator. Every collected data was checked daily by the supervisors and investigator for completeness.

### Data processing and analysis

Each questioner was checked for completeness, conciseness and clarity before data entry. Then the data were coded and entered using EpiData version 4.6 statistical software, exported to SPSS version 25 statistical software for data management and analysis. Data cleaning was done by removing duplicated data, invalid data, and correcting for spelling mistakes, and any error identified was corrected. The proportion of Maternal Birth Trauma with 95% CI was reported. For the determinant factors, the binary logistic regression model was fitted. Variables with p-value < 0.2 in the bivariable binary logistic regression analysis were considered for the multivariable binary logistic regression analysis. Multicollinearity diagnostics undertaken and there was no significant correlation between predictor variables. Hosmer and Lemeshow test for model fitness was checked. In the multivariable binary logistic regression, the Adjusted Odds Ratio (AOR) with 95% CI was reported to declare the statistical significance and strength of association. Finally, variables with p-value < 0.05 were used as cut of value to declare the statistical significance.

### Ethical considerations

#### Ethical approval

was obtained from the Institutional Ethical Review committee of School Of Medicine on behalf of the Institutional Review Board (IRB) of University of Gondar with reference number of SoM/1486/2022. A formal approval was sent and the permission for conducting the study was secured from the administration of UoGCSH and the head of the department of OBGYN before commencing the study. Personal identifiers were not used on the data collection proforma to ensure confidentiality of the study participants.

## Results

### Sociodemographic characteristics of study participants

In this study the response rate was 100% and out of 424 participants the mean age (± SD) was 26.83 years ± 5.220 years. Most of them were married, 412(97.2%), and were from Urban 365 (86.1%). Nearly two third of the participant, 259(61.1%), attained at least secondary school. More than half of them, 243 (57.3%), are house wives and quarter of the study participants, 109(25.7%), have monthly income less than 3000 ETB. Predominant position at work is standing and sitting (both) in two thirds, 276 (65.1%), of the participants (Table 1).

**Table 1:** Frequency distribution of Sociodemographic characteristics of mothers who gave birth vaginally at UoGCSH from 9th May to 9th August, 2022

Variables	Category	Frequency	Percentage
Age	< 21	58	13.7
	21–34	324	76.4
	≥ 35	42	9.9
Marital Status	Married	412	97.2
	Single and Divorced	12	2.8
Residence	Urban	365	86.1
	Rural	59	13.9
Level of Education	No formal Education	61	14.4
	Primary	104	24.5
	Secondary	154	36.3
	College and Above	105	24.8
Occupation`	Housewife	243	57.3
	Private employee	56	13.2
	Government employee	66	15.6
	Merchant and Others*	59	13.9
Predominant position at work	Standing	58	13.7
	Sitting	90	21.2
	Both**	276	65.1
Monthly Income	≤ 3000 ETB	109	25.7
	3001–5000 ETB	123	29
	5001–10,000 ETB	111	26.2
	> 10,000 ETB	81	19.1

*NGO, Student, job seeker; ** Standing and sitting

### Obstetric history of the study participants

According to this study nearly one third of the participants are primiparous 154(36.3%), and more than three fourth of them, 342(80.7%) have at least four ANC contacts (Table 2).

**Table 2:** Frequency distribution of obstetric related variables among mothers who gave birth vaginally at UoGCSH from 9th May to 9th August, 2022

Variable	Category	Frequency	Percentage
Parity	Primiparous	154	36.3
	Multiparous	270	63.7
Pregnancy Status	Planned	310	73.1
	Unplanned	114	26.9
Gestational Age	< 39 Weeks	126	29.7
	≥ 39 Weeks	298	70.3
Number of ANC contacts	< 4 times	82	19.3
	≥ 4 Times	342	80.7

### Delivery care and related variables

According to this study most of the study participants, 364(85.8%), have directed pushing in the second stage of labor. Maternal position during pushing was Lateral in about 4 out of 10 participants, 176(41.5%). Digital stretching of the perineum before crowning in more than half, 253 (59.7%) of the study participants. Majority of study participants delivered in Dorsolithotomy position, spontaneously and with perineum and/or fetal head supported in 413(97.4%), 406(95.8%) and 393(92.7%) respectively. Indicated episiotomy was done in one third, 140(33.0%) and fetal head position was occiput anterior in most, 369(87.0%) of the study participants. Total duration of labor was ≤ 3 h in few, 24(5.7%). Most of the study participants, 405(95.5%), delivered within two hours of second stage labor. Nearly two out of twelve, 71(16.7%) participants have Induction or Augmentation. More than half of the deliveries, 258(60.8%), were attended by midwives. About one fifth of the neonates, 90(21.2%), have birth weight ≥ 3500 g; more than half, 232(54.7%), were male. Nearly three fourth of the neonates, 317(74.8%) had head circumference greater than 35centimeter (Table 3).

**Table 3:** Frequency distribution of delivery care related variables among mothers who gave birth vaginally at UoGCSH from 9th May to 9th August, 2022

Variable	Category	Frequency	Percentage
Type pushing in the 2nd Stage	Directed	364	85.8
	Undirected	60	14.2
Maternal position during pushing	Lateral	176	41.5
	Dorsolithotomy	165	38.9
	Others**	83	19.6
Digital Stretching before crowning	Yes	253	59.7
	No	171	40.3
Maternal position during delivery	Lateral and Upright	11	2.6
	Dorsolithotomy	413	97.4
Type of delivery	Spontaneous	406	95.8
	Instrumental delivery	18	4.2
Delivery technique	Hands On/On	393	92.7
	Hands Off	31	7.3
Indicated Episiotomy done	Yes	140	33.0
	No	284	67.0
Fetal head position	Occiput anterior	369	87.0
	Occiput posterior	55	13.0
Total duration of labor	≤ 3 h	24	5.7
	3.1–12 h	313	73.8
	> 12 h	87	20.5
Duration of 2nd Stage	≤ 120 min	405	95.5
	> 120 min	19	4.5
Induction/Augmentation	Yes	71	16.7
	No	353	83.3
Birth Attendant	Midwife	258	60.8
	Physician	166	39.2
Birth weight (grams)	< 3500	334	78.8
	≥ 3500	90	21.2
Neonatal sex	Male	232	54.7
	Female	192	45.3
Neonatal head circumference (cm)	≤ 35	107	25.2
	> 35	317	74.8

***Supine, Upright

### Maternal outcome

**Fig. 2:**
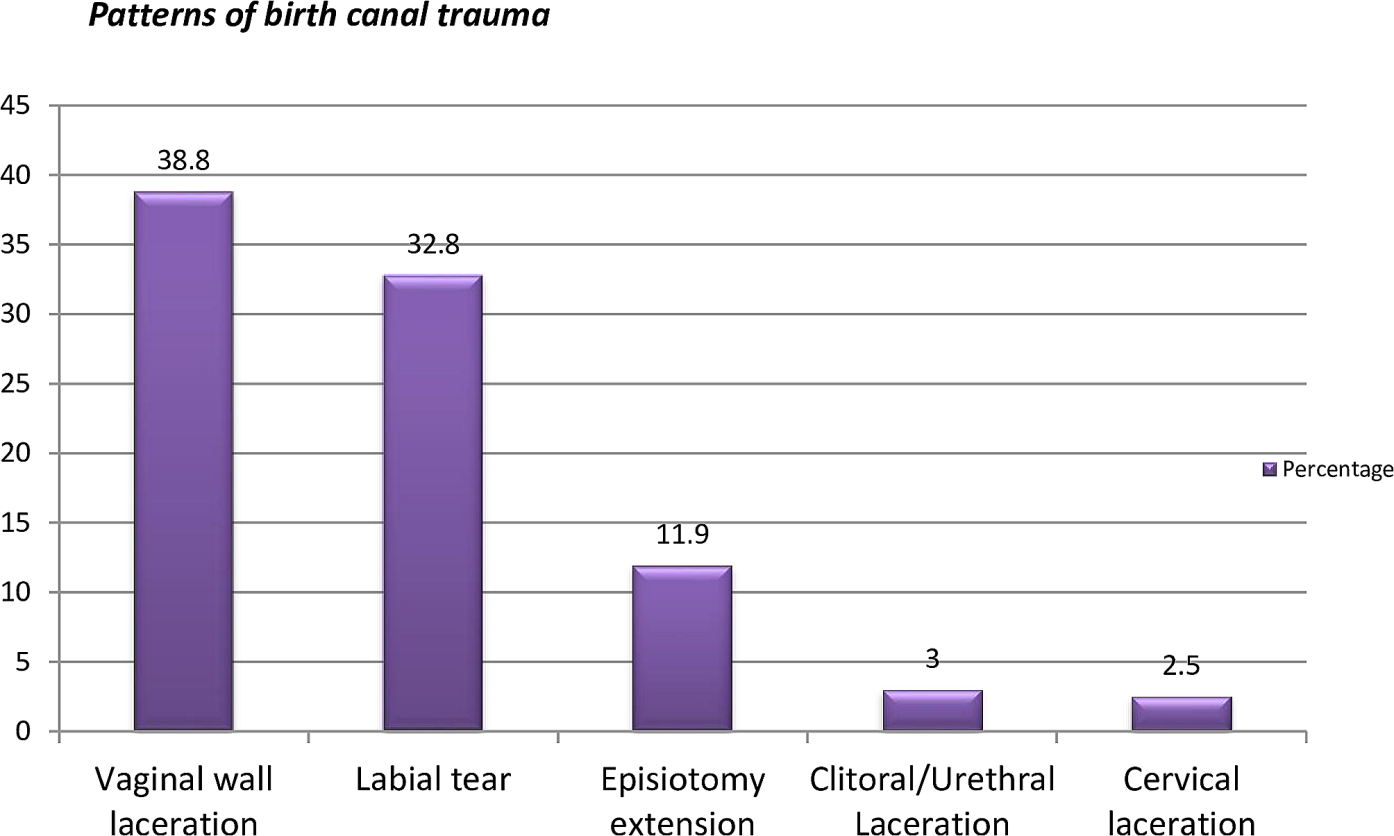
Patterns of birth canal injuries in women, who have maternal birth trauma after vaginal delivery at UoGCSH from 9th May to 9th August, 2022

From the total study participants nearly half of them, 201(47.4% with 95%CI 43.1, 51.7) have at least one form of Birth Trauma in the current childbirth. It was found to be 55.84% (86/154) in primiparous and 42.59% (115/270) in multiparous women. Among these about two fifth had vaginal wall laceration, 78 (38.8%), (Fig. 2).

**Fig. 3:**
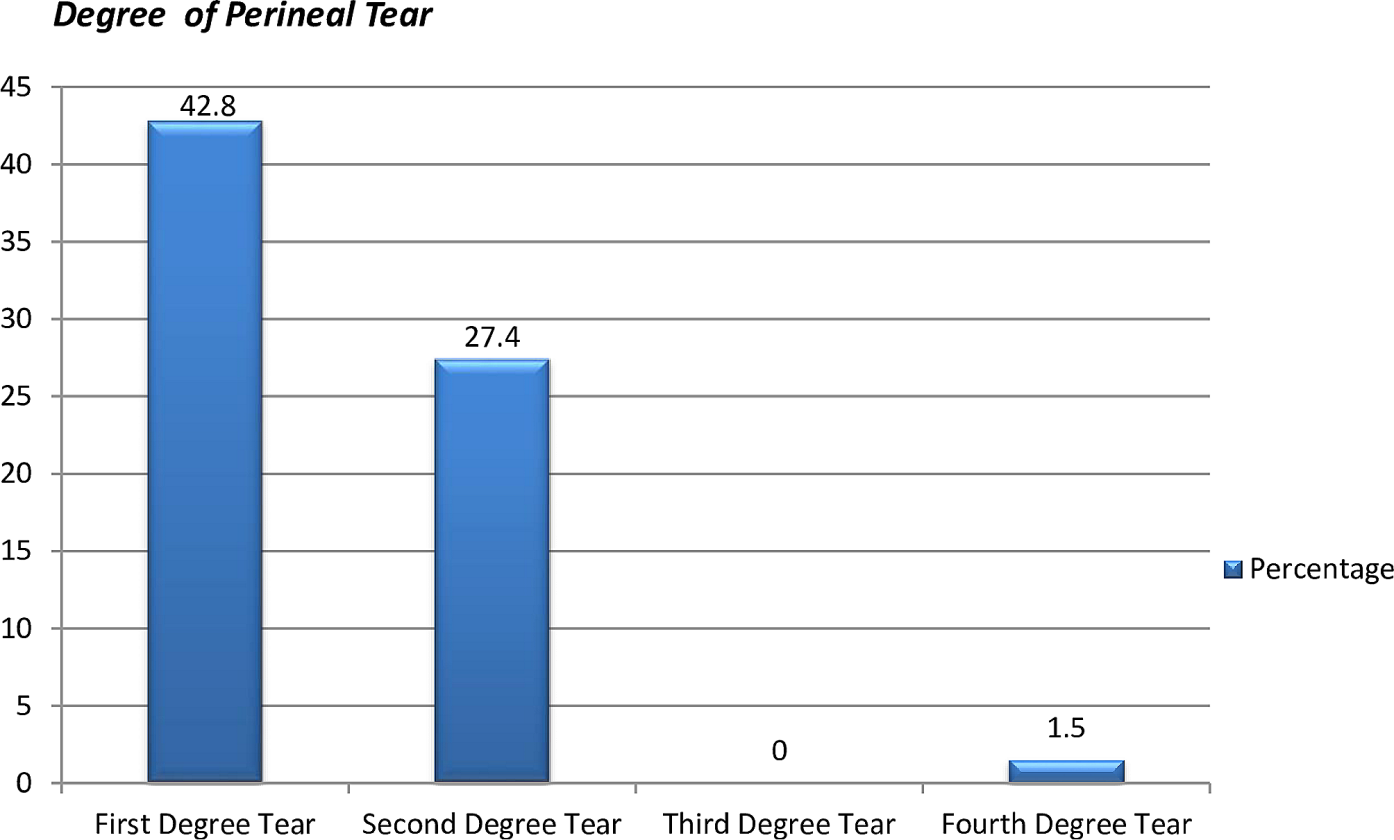
Degree of perineal tear among women, who have maternal birth trauma after vaginal delivery at UoGCSH from 9th May to 9th August, 2022

And it was observed from our result that first Degree perineal tear about, 85(42.3%) (Fig. 3).

#### Factors associated with maternal birth trauma

Marital status, Level of education, predominant work position, parity, gestational age, birth weight, neonatal sex, head circumference, type of delivery, delivery technique, total duration of labor and induction or augmentation of labor were identified as a candidate variable from bivariable logistic regression analysis and then fitted into the final multivariable logistic regression model using enter method to identify independent factors affecting maternal birth trauma. The odds of birth trauma among primiparous mothers were 3 times (AOR = 3.00; 95CI:1.68, 5.38) higher compared to multiparous mothers. The odds of birth trauma among women who gave birth at 39 weeks and later gestation were 2.96 times (AOR = 2.96; 95%CI:1.57, 5.57) higher compared to women who gave birth before 39 weeks. Mothers with delivery of 3500 g or more babies had 12.3 times (AOR = 12.3; 95%CI: 7.21, 40.1) higher odds of birth trauma as compared to mothers who delivered babies lower than 3500 g. The odds of birth trauma among mothers who gave birth babies with head circumference greater than 35 centimeter were 5.45 times (AOR = 5.45; 95%CI: 2.62, 11.31) higher than mothers who gave birth babies of head circumference not more than 35 centimeter. The odds of birth trauma among women with operative vaginal delivery were 6.59 times (AOR = 6.59; 95%CI: 1.44, 30.03) higher as compared to women with spontaneous delivery. Mothers who delivered without support to the perineum and/ or fetal head had 6.3 times (AOR = 6.30; 95%CI: 2.21, 17.94) higher odds of birth trauma as compared to mothers who delivered with the perineum and/or fetal head supported (Table 4).

**Table 4:** Factors affecting maternal birth trauma using multivariable logistic regression among mothers, who gave birth vaginally from 9th May to 9th August, 2022

Variable	Category	Birth Trauma		COR with 95%CI	AOR with 95%CI
		No (%)	Yes (%)		
Marital status	married	221(53.6)	191(46.4)	1	1
	Single, Divorced	2(16.7)	10(83.3)	5.78(1.25, 26.73)	3.67(0.61, 22.03)
Level of Education	No education	39(63.9)	22(36.1)	1	1
	Primary	56(53.8)	48(46.2)	1.51(0.79, 2.90)	1.45(0.57, 3.69)
	Secondary	79(51.3)	75(48.7)	1.68(0.91, 3.10)	1.54(0.63, 3.78)
	College	49(46.7)	56(53.3)	2.02(1.06, 3.87)	1.94(0.76, 4.97)
Work position	Standing	24(41.4)	34(58.6)	1.68(0.95, 2.99)	1.28(0.58, 2.82)
	Sitting	49(54.4)	41(45.6)	0.99(0.61, 1.60)	0.65(0.32, 1.29)
	Both	150(54.3)	126(45.7)	1	1
Parity	Primiparous	68(44.2)	86(55.8)	1.70(1.14, 2.54)	3.00(1.68, 5.38)**
	Multiparous	155(57.4)	115(42.6)	1	1
Gestational age	37-38w6d	93(73.8)	33(26.2)	1	1
	≥ 39	130(43.6)	168(56.4)	3.64(2.30, 5.76)	2.96(1.57, 5.57)**
Birth weight	< 3500	218(65.3)	116(34.7)	1	1
	≥ 3500	5(5.6)	85(94.4)	12.1(6.91,39.40)	12.3(7.21, 40.1) **
Sex	Male	112(48.3)	120(51.7)	1.46(0.99, 2.15)	1.11(0.65, 1.88)
	Female	111(57.8)	81(42.2)	1	1
Head circumference	<=35	92(86.0)	15(14.0)	1	1
	> 35	131(41.3)	186(58.7)	8.70(4.82, 15.70)	5.45(2.62, 11.31)***
Type of Delivery	Spontaneous	220(54.2)	186(45.8)	1	1
	OVD	3(16.7)	15(83.3)	5.91(1.68, 20.74)	6.59(1.44, 30.03)*
Delivery technique	Hands On	215(54.7)	178(45.3)	1	1
	Hands Off	8(25.8)	23(74.2)	3.47(1.51, 7.95)	6.30(2.21, 17.94)*
Total duration of labor	≤ 3 h	6(25.0)	18(75.0)	1.92(0.69, 5.33)	3.30(0.82, 13.20)
	3.1 to 12 h	183(58.5)	130(41.5)	0.45(0.28, 0.74)	0.54(0.27, 1.08)
	> 12 h	34(39.1)	53(60.9)	1	1
Induction or Augmentation	YES	25(35.2)	46(64.8)	2.35(1.38, 3.99)	0.498(0.23, 1.05)
	NO	198(56.1)	155(43.9)	1	1

## Discussion

In this study we tried to assess the burden of maternal birth trauma and its determinant factors. Maternal birth traumas following vaginal delivery are very common which contribute to significant maternal morbidity and even to death [1].

According to this study the overall prevalence of maternal birth trauma is 47.4% (95%CI: 43.1, 51.7). This is higher than a study done in South Africa which is 16.2% [9] and Brazil 38% [34] and lower than the study done in United Kingdom which is 70.01% [8], in Brazil 54% [35] and in Iran 84.3% [7]. This variation could be attributed due to the different group of study participants by race, set up of Obstetrics care and skill of care givers to detect perineal tear and episiotomy in these developed countries.

In our study, parity, gestational age, birth weight, head circumference and type of delivery were significantly associated with maternal trauma following vaginal delivery. The current study showed that prim parity have high odds of developing maternal perineal trauma. This is in line with most other studies from USA [36], Iran [7], United Kingdom [8], Brazil [35] and Uganda [37]. The possible reason could be due to the untested pelvis which was the first exposure to give birth physically and psychologically among primiparous women as compared to multiparous women [38].

This study showed that increased gestational age (≥ 39 weeks) at time of delivery significantly increases the risk of perineal trauma, which is consistent with a study done in Iran [7] and USA [39]. This could be attributed to the increased in birth weight in this category of participants along gestational age may cause shoulder dystocia and associated with maternal trauma following vaginal delivery [40].

The result of our study points out that heavier birth weight had significant association with perineal trauma. This is supported with other studies from USA [36], UK [8], German [18] and Uganda [37]. This is due to the bigger size of the fetus passing through the birth canal causes birth related trauma on the one hand and predictive avulsion and symptoms/signs of prolapse on the other hand compared to smaller babies [41].

Likewise, bigger head circumference had significant association with maternal birth trauma. This result is similar with studies from Iran [7], Brazil [34] and Australia [42]. This could be due to the larger presenting diameter of babies with bigger head circumference was linked to increase rate of obstetric anal sphincter injury among mothers following vaginal delivery as compared to babies with smaller head circumference.

According to the finding of this study, operative vaginal delivery had significant association with perineal trauma. This is in line with studies from Norway [43], United Kingdom [8], Brazil [34] and Boston, Massachusetts [44]. Moreover, our study showed that hands off fetal head and perineum delivery technique showed significant association with the odds of perineal trauma. This is inconsistent with the study from one review from Cochrane library which showed decreased episiotomy rate but this technique had no clear impact on other outcomes [45]. Another systematic review showed the incidence of third-degree lacerations and episiotomy rate increased with the hands-on technique [46]. This inconsistency could be due to warm compresses and Antepartum Perineal Massage (APM) routinely used which may decrease the risk of perineal tear which is totally lacking in our setup.

### Limitation of the study

We acknowledge the limitation of the current study design as it is institution based cross-sectional study. This study didn’t assess the long term effect of mothers with birth trauma.

## Conclusion

Maternal birth trauma following vaginal delivery was relatively high in this study. Prim parity, gestational age beyond 39 weeks at delivery, heavier birth weight, bigger head circumference, operative vaginal delivery and delivery without perineum and/or fetal head supported were factors affecting perineal outcome.

### Recommendations

**To Health Facilities including University of Gondar Hospital**

- Skilled birth attendants should do timely intervention like indicated episiotomy to avoid trauma to the perineum.
- Shared decision making between the patient and care provider of the likely complications of vaginal deliveries especially in mothers with macrosomic fetus.

**To Ministry of Health**

- Continuous professional training for skilled birth attends aimed at prevention of perineal trauma.
- Adoption of interventions such as warm compresses and antepartum perineal massage for improved perineal outcome in to the national management guideline and incorporation into practice.

**To researchers**

- Conduct of research including multiple centers with different level of care.
- Long term outcome of Obstetric perineal trauma shall be investigated.
- Since crossectional study design could not show cause effect relationship, it is recommended to use better study design for identifying the real cause of the problem.

## Data Availability

The data are available from the corresponding author on reasonable request.

## Abbreviations

ANC: Antenatal Care
AOR: Adjusted Odds Ratio
APM: Antepartum Perineal Massage
CD: Cesarean Delivery
CEmOC: Comprehensive Emergency Obstetric Care
CL: Cervical Laceration
EAS: External anal sphincter
HC: Head Circumference
IAS: Internal Anal Sphincter
ICI: International Consultation on Incontinence
MoH: Minster of Health
OASIs: Obstetric Anal Sphincter Injuries
OBGYN: Obstetrics and Gynecology
OPD: Outpatient Department
OVD: Operative Vaginal Deliveries
PPH: Postpartum Hemorrhage
RCOG: Royal College of Obstetricians and Gynecologists
SPSS: Statistical Package for Social Science
UI: Urinary Incontinence
UoGCSH: University of Gondar Comprehensive Specialized Hospital
UK: United Kingdom
USA: United States of America
VD: Vaginal Delivery and WHO: World Health Organization
